# The roles of OGT and its mechanisms in cancer

**DOI:** 10.1186/s13578-024-01301-w

**Published:** 2024-09-16

**Authors:** Xin Liu, Jing Wang, Yaoxian Xiang, Kangjie Wang, Dong Yan, Yingying Tong

**Affiliations:** Department of Oncology, Beijing Luhe Hospital Affiliated to Capital Medical University, Beijing, 101149 China

**Keywords:** OGT, O-GlcNACylation, Cancer-promoting factor, Drug resistance, Immune evasion

## Abstract

O-linked-N-acetylglucosaminylation (O-GlcNAcylation) is a common and important post-translational modification (PTM) linking O-linked β-N-acetylglucosamine (O-GlcNAc) to serine and threonine residues in proteins. Extensive research indicates its impact on target protein stability, activity, and interactions. O-linked N-acetylglucosamine transferase (OGT) is a critical enzyme that catalyzes O-GlcNAc modification, responsible for adding O-GlcNAc to proteins. OGT and O-GlcNAcylation are overexpressed in many tumors and closely associated with tumor growth, invasion, metabolism, drug resistance, and immune evasion. This review delineates the biochemical functions of OGT and summarizes its effects and mechanisms in tumors. Targeting OGT presents a promising novel approach for treating human malignancies.

## Introduction

O-linked-N-acetylglucosaminylation (O-GlcNAcylation), an important protein glycosylation modification, has its origins in the earliest report by Hart’s team [1, 2]. Subsequently, researchers defined O-GlcNAcylation as the process of adding O-linked β-N-acetylglucosamine (O-GlcNAc) to serine or threonine residues in proteins [3]. This modification is implicated in various cellular processes, such as signal transduction, cell cycle regulation, transcriptional control, and metabolism [4–7].

Roughly 2–5% of glucose feeds into the hexosamine biosynthetic pathway (HBP) to generate UDP-GlcNAc, the sugar donor for O-GlcNAcylation [8]. The key rate-limiting enzyme in the HBP pathway is glutamine–fructose-6-phosphate amido transferase (GFAT), which converts fructose-6-phosphate into glucosamine-6-phosphate [9]. O-GlcNAcylation undergoes dynamic and reversible regulation through O-GlcNAc addition and removal. O-GlcNAc transferase (OGT) attaches O-GlcNAc to Ser/Thr residues of substrate proteins, while O-GlcNAcase (OGA) is responsible for its cleavage [10].

The gene responsible for encoding *OGT* resides on the X chromosome [11]. The OGT protein consists of an N-terminal tetratricopeptide-repeats (TPRs) domain, which binds substrate proteins, and a C-terminal catalytic domain that catalyzes substrate O-GlcNAcylation [12, 13]. Moreover, OGT participates in diverse physiological processes, including fostering nervous system development, regulating mammalian cell physiology, and preserving hematopoietic stem cells. Its expression is elevated in various tumors, suggesting a role in tumor promotion. This review outlines the biochemical functions of OGT and summarizes its role and specific mechanisms in tumors, aiming to provide new insights and approaches for treating malignant tumors.

## Structure and basic function of OGT

OGT, a member of the GT-B glycosyltransferase family, is responsible for attaching O-GlcNAc to substrate proteins [14, 15]. OGT is highly conserved across various organisms, from Caenorhabditis elegans to mammals [16]. In humans, alternative splicing produces three OGT isoforms: nucleocytoplasmic OGT (ncOGT), mitochondrial OGT (mOGT), and short OGT (sOGT), each differing in location and length [17]. These isoforms are encoded by the same gene on the X chromosome and feature N-terminal TPRs and a multi-domain catalytic C-terminal [18]. The primary structural difference between the three OGT isoforms is the number of N-terminal TPRs. The longest isoform, ncOGT, contains 13.5 TPRs, while mOGT and sOGT contain 9.5 and 2.5 TPRs, respectively [19, 20]. TPR sequences are primarily involved in the recognition and binding of substrate proteins by OGT [21, 22]. These sequences fold into an antiparallel α-helical structure, with adjacent repeats forming a superhelical structure that binds specific substrates [23, 24]. The enzymatic domain of OGT is located at the C-terminus and catalyzes the O-GlcNAcylation of substrate proteins [12]. This modification affects protein stability, conformation, localization, and activity [25–29]. To date, thousands of proteins have been identified as O-GlcNAcylation targets, including transcription factors, membrane proteins, and cytoskeletal proteins. These modifications regulate gene transcription, cellular responses, protein translation, protein degradation, and other critical biological processes. O-GlcNAcylation impacts cell signal transduction and plays a crucial regulatory role in normal growth and development, as well as in the pathogenesis of various diseases [30–33].

## OGT in cancer progression

### Roles of OGT in cancer proliferation

Abnormal proliferation is a hallmark of cancer. The carcinogenic effect of OGT is closely associated with its role in driving cell growth in various malignancies, such as liver cancer, gastric cancer (GC), and colorectal cancer (CRC). [34–36]. OGT promotes tumor proliferation primarily through its involvement in regulating protein post-translational modifications (PTMs).

**Fig. 1:**
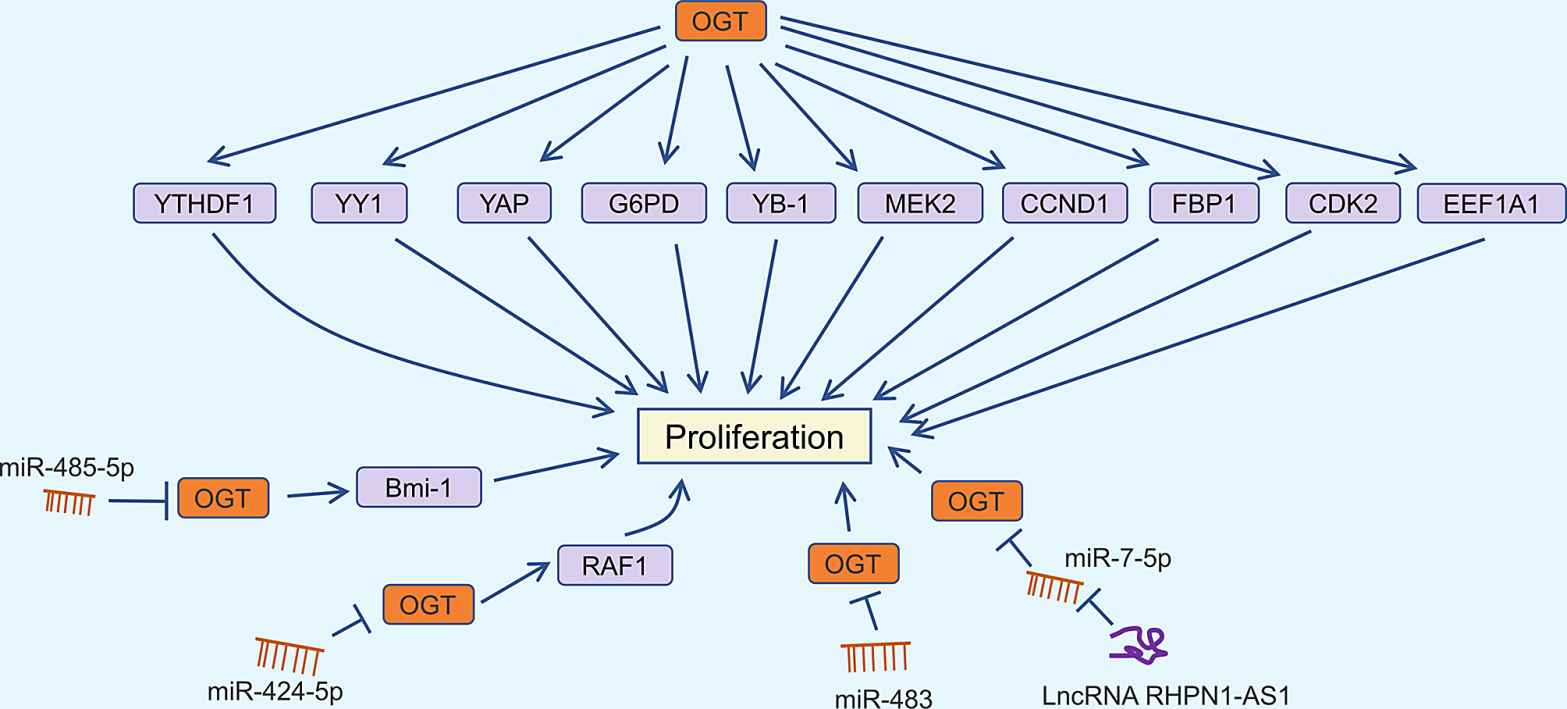
OGT and proliferation regulation in cancer

In non-small cell lung cancer cells, OGT overexpression following glutamine deprivation abolishes fructose-1,6-bisphosphatase 1 (FBP1) phosphorylation and enhances β-oxidation gene transcription via FBP1 O-GlcNAcylation, thus promoting cell proliferation [37]. Similar findings have been observed in hepatocellular carcinoma (HCC), highlighting OGT’s crucial role in tumor growth by regulating FBP1 [38]. Additionally, Y box binding protein 1 (YB-1), a well-known oncoprotein, is associated with tumor immune evasion and drug resistance [39, 40]. Liu et al. demonstrated that OGT increases O-GlcNAcylation of YB-1 at Thr126, thereby promoting cell proliferation in HCC [41]. Targeting OGT significantly impedes the progression of high-fructose-induced HCC, with the O-GlcNAcylation of eukaryotic elongation factor 1A1 (EEF1A1) playing a pivotal role in this process [42]. Mitogen-activated protein kinase kinase 2 (MEK2), an important molecule in the MAPK signaling pathway, is related to cell proliferation, differentiation, and stress response [43]. OGT promotes the stability of MEK2 through O-GlcNAcylation at Thr13, thereby enhancing the proliferation and migration of breast cancer cells [44]. Furthermore, microRNA-485-5p modulates CRC proliferation by regulating the stability of B-cell-specific Moloney murine leukemia virus integration region 1 (Bmi-1) via OGT [45]. In xenograft models, mutating the O-GlcNAcylation site of YTH domain family 1 (YTHDF1) reduced tumor growth [46]. DNA polymerase iota (Pol ι) activates glucose-6-phosphate dehydrogenase (G6PD) through Erk-OGT-induced O-GlcNAcylation, promoting the proliferation of esophageal squamous cell carcinoma [47]. Yu et al. found that miR-483 targets OGT to inhibit the proliferation of GC cells [48]. Moreover, the X-inactive-specific transcript (XIST)/miR-424-5p/OGT axis regulates RAF1 glycosylation, impacting liver cancer growth [49]. Long non-coding RNA RHPN1-AS1 is significantly upregulated in CRC cell lines, facilitating CRC progression by modulating the miR-7-5p/OGT axis [50]. These findings indicate that OGT is a key regulator of tumor proliferation (Fig. 1).

### Roles of OGT in cancer invasion and metastasis

Metastasis, the spread and growth of tumor cells from their original site to new locations in the body, is the leading cause of cancer-related deaths [51]. Despite extensive research on invasion and metastasis mechanisms, this regulatory process remains poorly understood. Recent studies have identified OGT as a key regulator of tumor invasion and metastasis.

Epithelial-mesenchymal transition (EMT) is the phenotypic change of cells from an epithelial to a mesenchymal state, resulting in increased motility and invasiveness of tumor cells [52]. EMT is characterized by the loss of epithelial cell-cell connections, cytoskeletal reorganization, decreased expression of E-cadherin, and increased N-cadherin [53]. Numerous studies have explored EMT’s role in promoting tumor cell invasion and malignancy. Jiang et al. found that knocking down enhancer of zeste homolog 2 (EZH2) in colorectal cancer partially reverses the EMT changes induced by OGT-mediated O-GlcNAcylation [54]. Additionally, OGT knockdown has been shown to inhibit the expression of EMT markers (N-cadherin and Slug), migration, and invasion in lung cancer cells, with the interaction between OGT and STAT3 playing a crucial role in this process [55]. Moreover, OGT knockdown in HO-8910PM cells resulted in decreased O-GlcNAcylation and increased expression of E-cadherin [56].

Matrix metalloproteinases (MMPs), members of the metzincin protease superfamily, degrade the extracellular matrix (ECM) [57]. MMPs participate in physiological processes such as embryonic development and wound healing and play a vital role in enhancing tumor cell migration and invasion [58, 59]. OGT regulates matrix metalloproteinase levels, thereby affecting tumor metastasis. Qiao et al. found that suppressing OGT weakened the migration ability of esophageal cancer cells by significantly reducing the expression of matrix metalloproteinase 9 (MMP9) in Eca-109 cells [60]. Furthermore, OGT knockdown in prostate cancer cell lines was associated with reduced expression of MMP-2, MMP-9, and vascular endothelial growth factor (VEGF), thereby inhibiting invasion and angiogenesis through the regulation of the oncogenic transcription factor forkhead box M1 (FoxM1) [61].

**Fig. 2:**
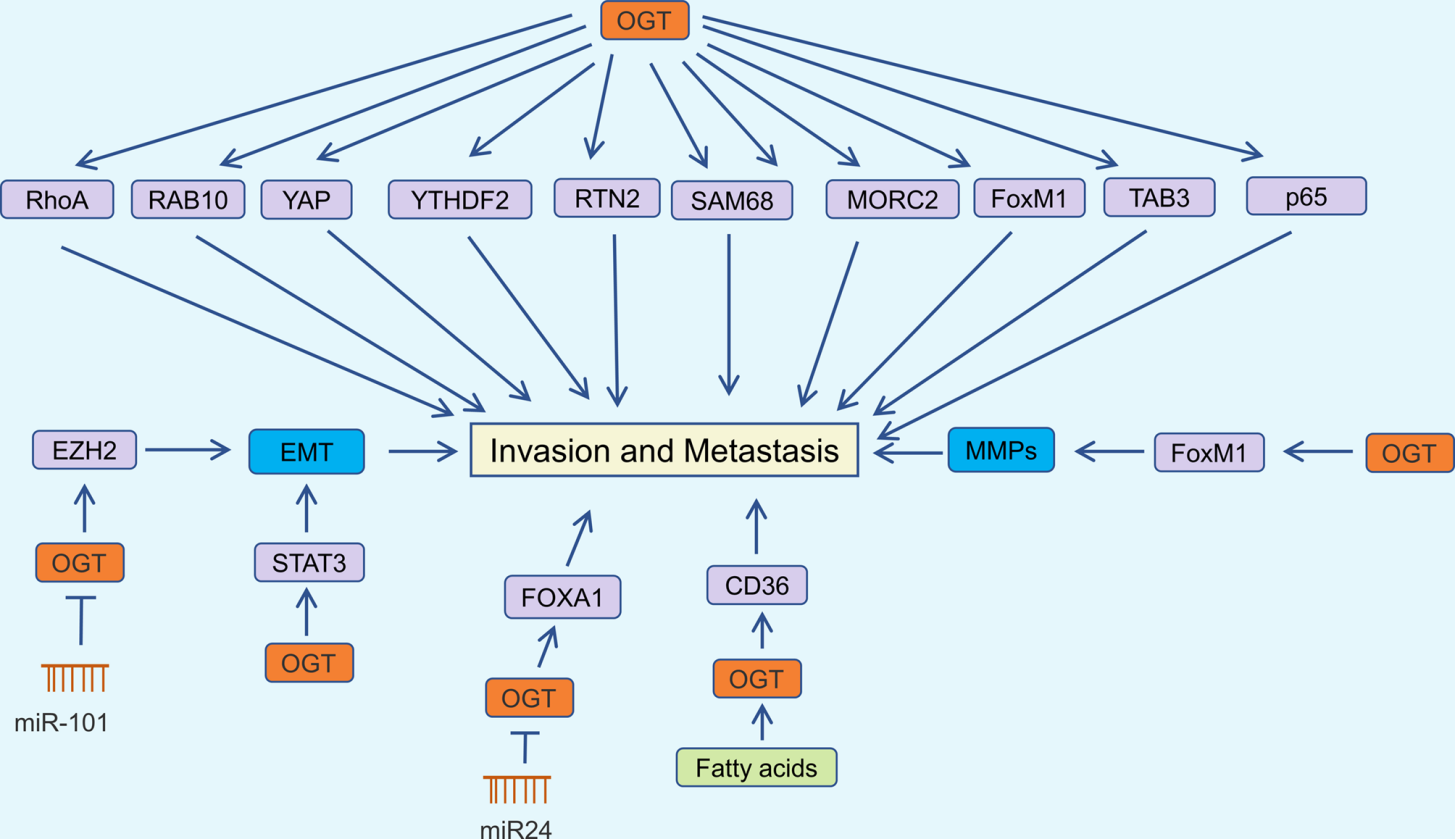
OGT in cancer invasion and metastasis regulation

OGT orchestrates O-GlcNAcylation to drive the migration and invasion of papillary thyroid cancer by activating Yes-associated protein (YAP) at the Ser109 modification site [62]. Lv et al. identified upregulated OGT expression in HCC, demonstrating its role in promoting tumor aggressiveness through OGT-mediated O-GlcNAcylation, which stabilizes ras-related protein Rab-10 (RAB10) [63]. CD36, a cell membrane protein, mediates fatty acid uptake and is associated with fatty acid absorption in the heart, skeletal muscle, and adipose tissue [64, 65]. Jiang et al. confirmed that fatty acids promote gastric cancer metastasis by inducing CD36 expression via OGT-mediated O-GlcNAcylation [66]. YTH N6-methyladenosine RNA binding protein 2 (YTHDF2) plays a crucial role in N6-methyladenosine (m6A) modification, regulating mRNA degradation [67, 68]. OGT promotes hepatitis B virus-related HCC migration and invasion by mediating O-GlcNAcylation of YTHDF2 at Ser263 [69]. Wang et al. found that reticulon 2 (RTN2) interacts with OGT and is modified by O-GlcNAc; inhibiting OGT abolishes the stimulatory effects of RTN2 on cell migration [70]. SRC-associated in mitosis of 68 kDa (SAM68) is O-GlcNAcylated and predominantly interacts with OGT in the nucleus, promoting lung cancer cell migration and invasion [71]. Multiple studies have linked MORC family CW-type zinc finger 2 (MORC2) with DNA damage and resistance to radiotherapy and chemotherapy in breast cancer [72, 73]. Liu et al. found that OGT O-GlcNAcylates MORC2 at Thr556, thereby promoting breast cancer migration, invasion, and metastasis [74]. Reginato’s team demonstrated that reducing OGT expression significantly decreases FoxM1 protein levels, inhibiting breast cancer cell growth and invasion [75]. Non-coding RNAs also regulate breast cancer progression by altering OGT expression. Inhibition of OGT by miR-24 reduces the stability of forkhead box protein A1 (FOXA1), thereby inhibiting breast cancer cell invasion [76]. Overexpression of OGT significantly enhances O-GlcNAcylation in TAK1 binding protein 3 (TAB3), promoting migration and invasion of triple-negative breast cancer (TNBC) cells in vivo and in vitro [77]. Dysregulation of the NF-κB pathway is increasingly recognized as a key regulator of tumor progression and drug resistance [78, 79]. Ali et al. confirmed that OGT knockdown reduced CXCR4 expression by decreasing O-GlcNAcylation of NF-κB p65 (p65), inhibiting cervical cancer metastasis [80]. Niu et al. showed that OGT-mediated O-GlcNAcylation regulates ras homolog family member A (RhoA) activity in ovarian cancer cells, affecting their migration and invasion [81]. These studies indicate that OGT overexpression significantly promotes tumor cell invasion into other tissues, facilitating their survival and cancer spread. Therefore, targeting OGT may help inhibit tumor metastasis (Fig. 2) [82–86].

### Roles of OGT in cancer metabolism

Abnormal cancer metabolism plays a crucial role in tumorigenesis, metastasis, and drug resistance [87]. Glucose, lipid, and amino acid metabolism in tumor tissue undergo significant changes compared to normal tissue [88]. Studies have shown that OGT is directly or indirectly involved in the regulation of tumor metabolic processes.

Most tumor cells produce adenosine triphosphate (ATP) primarily through glycolysis, even under adequate oxygen levels. This phenomenon, known as the Warburg effect, supports tumor cell growth [89]. Phosphoglycerate kinase 1 (PGK1) is the first ATP-generating enzyme in glycolysis, and its expression is linked to tumor progression [90–92]. Research has shown that OGT overexpression enhances PGK1 activity by increasing its O-GlcNAcylation. Blocking T255 O-GlcNAcylation of PGK1 inhibits glycolysis, enhances the mitochondrial tricarboxylic acid (TCA) cycle, and suppresses colon cancer growth [93]. Additionally, transient expression of wild-type OGT increases PKM2 O-GlcNAcylation, suppresses pyruvate kinase activity in HeLa cells, stimulates aerobic glycolysis, and promotes tumor growth [94]. OGT-mediated O-GlcNAcylation also enhances the stability of isocitrate dehydrogenase 2 (IDH2) protein, thereby activating the NF-κB signaling pathway, reprogramming glucose metabolism, and promoting CRC progression [95].

Reprogramming lipid metabolism is a hallmark of many malignancies. Increased fat uptake and lipogenesis occur in various cancers, leading to rapid tumor growth. Lipids form the basic structure of membranes and also serve as signaling molecules and energy sources [96, 97]. Sterol regulatory element-binding protein 1 (SREBP-1) is a major transcription factor controlling lipid metabolism and a key link between oncogenic signaling and tumor metabolism [98]. OGT regulates the expression of SREBP-1 in a proteasomal and AMP-activated protein kinase (AMPK)-dependent manner, thereby altering lipid metabolism and impacting breast cancer cell survival [99].

Acetyl-CoA, produced by acetyl-CoA synthetase 2 (ACSS2) through the catalysis of acetate, is crucial for tumor growth and survival [100, 101]. OGT has been shown to regulate glioblastoma acetate metabolism by influencing cyclin-dependent kinase 5 (CDK5)-dependent ACSS2 phosphorylation. Moreover, drugs targeting OGT and CDK5 have demonstrated efficacy in reducing glioblastoma tumors in vitro [102]. Therefore, OGT plays a significant role in the metabolic reprogramming of tumors and represents a promising therapeutic target (Table 1).

**Table 1:** Roles of OGT in cancer metabolism

Molecular pathway	Function	References
OGT/PGK1	Promotes the glycolysis and growth; Inhibit mitochondrial TCA cycle	[93]
OGT/PKM2	Promotes the aerobic glycolysis and growth	[94]
OGT/IDH2	Promotes the proliferation and lactic acid production; Reduces ROS production	[95]
OGT/SREBP-1	Promotes the cell survival and lipid synthesis	[99]
OGT/CDK5	Regulates acetate metabolism	[102]

### Roles of OGT in drug resistance

Due to the high morbidity and mortality associated with tumors, significant efforts have been made to develop anticancer drugs. These drugs play a crucial role in inhibiting tumor cell metastasis and reducing tumor cell survival. However, tumor cells can alter multiple molecular pathways to develop drug resistance [103, 104].

Platinum-based drugs are commonly used in the treatment of ovarian cancer (OC), but the development of drug resistance remains a significant challenge [105]. Reducing OGT-mediated O-GlcNAcylation of synaptosome-associated protein-23 (SNAP-23) promotes cisplatin resistance by inducing exosome secretion in OC [106]. Additionally, the downregulation of OGT, leading to reduced O-GlcNAcylation of synaptosome-associated protein-29 (SNAP-29), enhances cisplatin-induced autophagy, making OC cells less responsive to cisplatin treatment [107]. Huang et al. demonstrated that OGT interacts with kelch-like ECH-associated protein 1 (KEAP1) and promotes its glycosylation in A2780 and A2780/DDP cell lines. Furthermore, miR-181d enhances OC resistance to cisplatin by regulating the OGT/KEAP1/Nrf2 axis both in vitro and in vivo [108].

5-fluorouracil (5-FU) is a crucial drug for treating colorectal cancer, targeting thymidylate synthase (TS) and its metabolites [109, 110]. Very et al. showed that TS undergoes O-GlcNAcylation through its interaction with OGT, which impedes proteasomal degradation and enhances its stability. Knockdown of OGT reduced cancer cell sensitivity to 5-FU by lowering both TS protein levels and activity [111].

Proteasome inhibitors are used to treat multiple myeloma and mantle cell lymphoma [112]. Inhibition of OGT in NCI-H460 cells and their xenograft model increases cancer cell sensitivity to proteasome inhibitors. This effect is due to the stabilization of nuclear factor erythroid 2-related factor 1 (NRF1) via OGT-catalyzed O-GlcNAcylation, leading to the upregulation of proteasome subunit genes [113].

Osteosarcoma, a common primary bone tumor, shows a reduced survival rate post-metastasis [114]. Methotrexate is an approved therapeutic drug for osteosarcoma [115]. Sun et al. identified that lncRNA EBLN3P increases the resistance of osteosarcoma cells to methotrexate by enhancing the miR-200a-3p/OGT axis [116]. Additionally, docetaxel is a chemotherapy drug approved for prostate cancer treatment [117]. Xia et al. confirmed that miR-140 induces prostate cancer cell sensitivity to docetaxel in an OGT-dependent manner. Knockdown of OGT sensitizes prostate cancer cells to docetaxel [118]. Cisplatin-based systemic chemotherapy is the standard treatment for advanced bladder cancer [119]. Wang et al. observed that reducing OGT expression increased bladder cancer cell sensitivity to cisplatin [120]. Furthermore, gemcitabine and paclitaxel are also used in bladder cancer treatment [121]. OGT knockdown significantly enhanced the sensitivity of drug-resistant bladder cancer cells to these chemotherapy drugs [122].

Based on these studies, OGT-mediated O-GlcNAcylation is associated with tumor treatment resistance. However, current research on targeting OGT for overcoming drug resistance is still limited. This aspect should be further explored in future studies.

### Roles of OGT in apoptosis

**Fig. 3:**
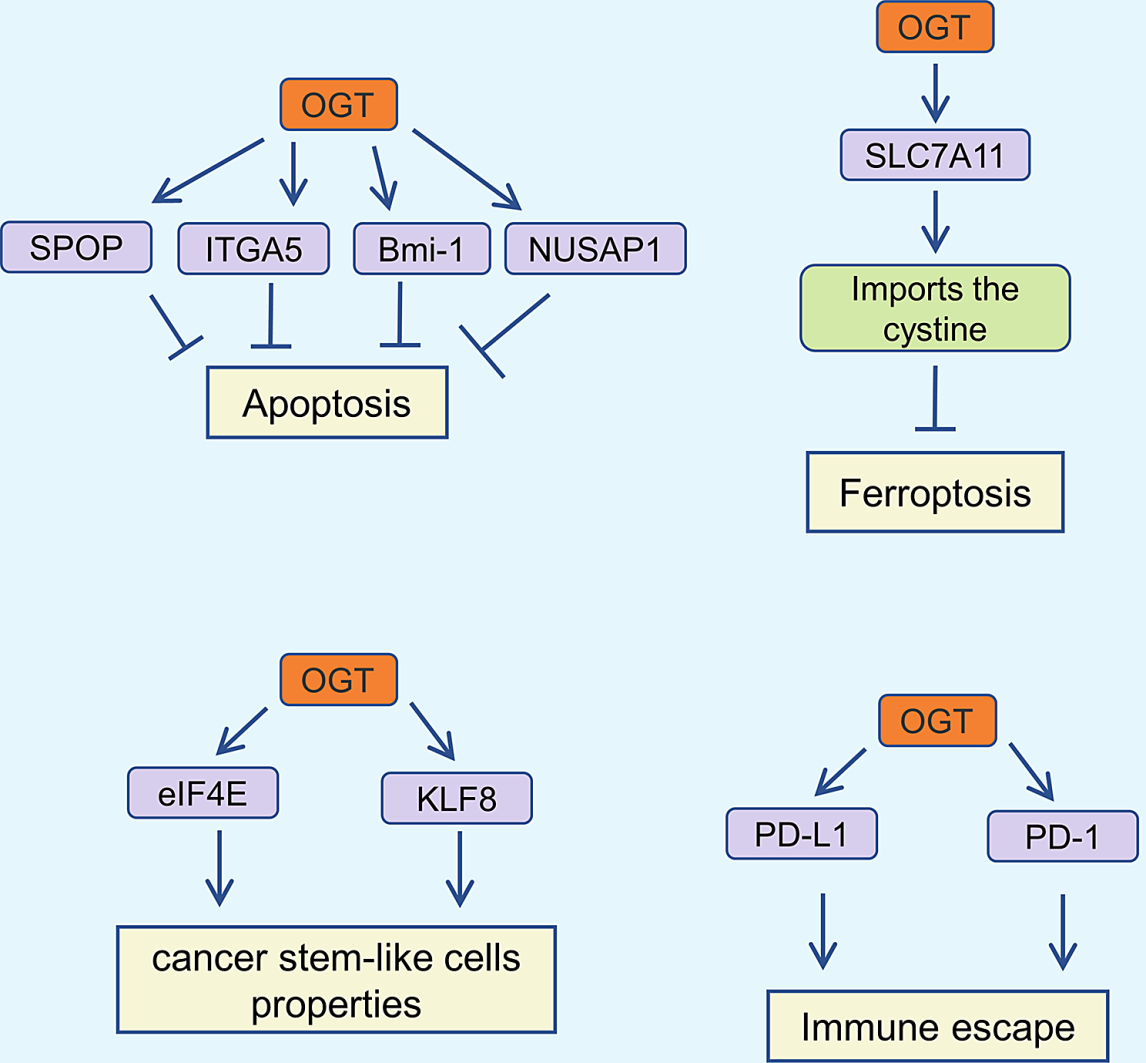
Roles of OGT in the regulation of apoptosis, cancer stem-like cells properties, ferroptosis and immune escape in cancer

Apoptosis, first defined as a programmed cell death mode in 1972, is characterized by nuclear and chromatin condensation, as well as the formation of apoptotic bodies, which can be observed through light microscopy [123–125]. This process is crucial for maintaining homeostasis in normal tissues, including the gastrointestinal tract, immune system, and skin [126, 127]. However, abnormal apoptosis occurs during tumor progression, leading to reduced apoptosis in tumor cells and enhanced survival [128]. Given OGT’s role in promoting tumor progression, studies have shown that it inhibits tumor cell apoptosis. For instance, in HCC, OGT highly O-GlcNAcylates speckle-type POZ protein (SPOP) at Ser96, facilitating its nuclear entry and inhibiting apoptosis of liver cancer cells [129]. Yu et al. discovered that OGT-mediated O-GlcNAcylation influences integrin α5 (ITGA5) protein stability, promoting tumor cell growth and tumorigenesis while reducing apoptosis [130]. Additionally, OGT O-GlcNAcylates Bmi-1 at Ser255, thus inhibiting apoptosis in prostate cancer cells [131]. Nuclear and spindle-associated protein 1 (NUSAP1) has been shown to promote bladder cancer progression through the TGF-β signaling pathway, and its expression is also associated with lymph node metastasis and survival prognosis [132, 133]. The protein stability of NUSAP1 decreases after knocking down OGT expression in HT-1376 and T24 cells to reduce O-GlcNAcylation, thus promoting bladder cancer cell apoptosis [134]. Therefore, targeting OGT may be advantageous in inducing tumor cell apoptosis (Fig. 3).

### Roles of OGT in cancer stem-like cells properties

Tumor initiation and progression are regulated by cancer stem cells (CSCs), which possess self-renewal, plasticity, and differentiation capabilities that promote metastasis, drug resistance, and recurrence [135, 136]. Eukaryotic initiation factor 4E (eIF4E) and RAF proto-oncogene serine/threonine-protein kinase (RAF1) are key targets of sorafenib [137, 138]. Research has shown that OGT enhances the stem cell-like potential of HCC cells by upregulating eIF4E [139]. Additionally, OGT establishes a feedback loop with Krüppel-like factor 8 (KLF8), modulating CSC phenotypes and increasing paclitaxel resistance [140]. Therefore, identifying the signaling networks that regulate CSC properties via OGT could contribute to more effective tumor treatments (Fig. 3).

### Roles of OGT in ferroptosis

Ferroptosis is a recently identified mode of cell death with unique properties and functions, implicated in various diseases, including tumors, renal disease, and cardiovascular disease [141–143]. It is primarily characterized by cytological changes such as the reduction or disappearance of mitochondrial cristae and the condensation of mitochondrial membranes [144, 145]. Recent studies have shown that OGT is associated with ferroptosis in tumors.

Solute carrier family 7, member 11 (SLC7A11), a cystine/glutamate antiporter, promotes cystine import into cells, thereby inhibiting lipid peroxidation and ferroptosis [146, 147]. It has been reported that OGT promotes cystine uptake by HCC cells through O-GlcNAcylation of SLC7A11 at the Ser26 site, leading to ferroptosis inhibition in HCC [148]. Hypoxia-inducible factor 2α (HIF-2α), a hypoxia-related transcription factor, promotes renal cancer progression [149]. OGT was found to increase HIF-2α protein levels in clear cell renal cell carcinoma by inhibiting ubiquitin-proteasome-mediated degradation. Moreover, the OGT/HIF-2α axis modulates the sensitivity of clear cell renal cell carcinoma to ferroptosis [150]. These findings indicate that OGT plays a crucial role in the ferroptosis process, although the regulatory mechanism requires further investigation (Fig. 3).

### Roles of OGT in autophagy

Autophagy is a self-degradative process essential for maintaining cellular homeostasis under stress conditions [151, 152]. Well-known regulatory pathways of autophagy include AMPK, PI3K/Akt/mTOR, and Beclin-1 [153]. Recent studies have highlighted the role of OGT in autophagy. Jin et al. demonstrated that overexpression of OGT in bladder cancer cells increases the O-GlcNAcylation level of AMPKα, resulting in altered autophagy flux [154]. Further research is required to elucidate the interaction between OGT and autophagy-related molecules and to characterize its role in tumor progression.

### Roles of OGT in immune escape

Since the early 20th century, researchers have explored the role of the immune system in tumor development. Tumor immunosurveillance is a critical process where the immune system monitors, identifies, and eliminates tumor cells [155, 156]. Immune checkpoint proteins, such as Programmed Death-Ligand 1 (PD-L1) and its receptor PD-1, are closely related to tumor immune evasion. PD-L1 interacts with PD-1 on cytotoxic T lymphocytes, transmitting inhibitory signals that weaken the tumor-killing function of these cells [157, 158]. Recent studies have reported that OGT is involved in regulating the expression of immune checkpoint proteins. OGT has been implicated in promoting tumor immune evasion by inhibiting the lysosomal degradation of PD-L1 [159]. Yuan et al. demonstrated that exosomal OGT enhances immune evasion of esophageal cancer stem cells by upregulating PD-1 expression in CD8^+^ T cells [160]. These findings indicate that OGT plays a role in tumor immune evasion. Further research is needed to determine whether OGT can regulate other immune checkpoint molecules (Fig. 3).

## Targeting OGT and O-GlcNAcylation

In most human tumors, OGT functions as an oncoprotein, promoting tumor growth, metastasis, and drug resistance by activating signaling pathways such as proliferation, EMT, and anti-apoptosis. Therefore, targeting OGT is a promising strategy for cancer treatment. For instance, quercetin has been reported to induce cell death in cervical cancer by reducing the expression of OGT, overall O-GlcNAc, and O-GlcNAcylated AMPK [161]. Similarly, corosolic acid inhibits liver cancer progression by decreasing OGT expression and O-GlcNAcylation levels in cancer cells [162]. A number of small molecule compounds targeting OGT activity or OGT-mediated O-GlcNAcylation have been produced, such as OSMI-1 and OSMI-4, and have been widely used in tumor research [163, 164]. OSMI-1 significantly inhibits the proliferation and migration of thyroid cancer cells and slows the occurrence of liver tumors [62, 69].

Moreover, combination therapies have proven more effective than monotherapies in treating tumors. For example, astragalus polysaccharide reduces OGT levels and increases OGA levels in liver cancer cells, thereby downregulating O-GlcNAcylation and promoting doxorubicin-induced apoptosis [165]. A notable increase in cell death has been observed with the coadministration of OSMI-1 and temozolomide. These findings highlight OGT as a promising drug target. However, many phytochemicals and small molecule inhibitors face challenges such as low bioavailability and solubility in human applications. Future applications could overcome these limitations through the use of nanomaterials [166].

## Conclusion and perspectives

This review provides an overview of the biological functions of OGT, with a particular focus on its impact on tumors. Elevated levels of OGT expression are commonly observed in tumors. OGT-mediated O-GlcNAcylation promotes tumor cell proliferation and induces EMT, facilitating MMP expression, which is associated with tumor invasion and metastasis. Tumor metabolic reprogramming is also linked to OGT, thereby influencing tumor progression. Furthermore, the OGT/O-GlcNAcylation pathway inhibits apoptosis and ferroptosis, promotes tumor immune escape, and ultimately enhances tumor growth. Notably, OGT mediates drug resistance, making it a critical target for altering tumor cell sensitivity to anticancer treatments. Consequently, small molecule compounds have been developed to inhibit the OGT/O-GlcNAcylation pathway. Knockdown of OGT has been shown to reduce malignant tumor behavior both in vitro and in vivo. Future efforts should focus on translating these research findings into clinical applications to improve cancer patient outcomes.

## Data Availability

Not applicable.
